# WSCNet: Biomedical Image Recognition for Cell Encapsulated Microfluidic Droplets †

**DOI:** 10.3390/bios13080821

**Published:** 2023-08-15

**Authors:** Xiao Zhou, Yuanhang Mao, Miao Gu, Zhen Cheng

**Affiliations:** Department of Automation, Tsinghua University, Beijing 100084, China

**Keywords:** droplet microfluidics, convolutional neural network (CNN), single-cell encapsulation, image recognition

## Abstract

Microfluidic droplets accommodating a single cell as independent microreactors are frequently demanded for single-cell analysis of phenotype and genotype. However, challenges exist in identifying and reducing the covalence probability (following Poisson’s distribution) of more than two cells encapsulated in one droplet. It is of great significance to monitor and control the quantity of encapsulated content inside each droplet. We demonstrated a microfluidic system embedded with a weakly supervised cell counting network (WSCNet) to generate microfluidic droplets, evaluate their quality, and further recognize the locations of encapsulated cells. Here, we systematically verified our approach using encapsulated droplets from three different microfluidic structures. Quantitative experimental results showed that our approach can not only distinguish droplet encapsulations (F1 score > 0.88) but also locate each cell without any supervised location information (accuracy > 89%). The probability of a “single cell in one droplet” encapsulation is systematically verified under different parameters, which shows good agreement with the distribution of the passive method (Residual Sum of Squares, RSS < 0.5). This study offers a comprehensive platform for the quantitative assessment of encapsulated microfluidic droplets.

## 1. Introduction

Droplet-based microfluidic platforms have been broadly adopted in various biotechnology applications, such as directed evolution, single-cell sequencing, and digital PCR, owing to their high-throughput capacity and single-molecule sensitivity. In addition, its miniaturization offers great advantages due to a larger surface-to-volume ratio, which confers reproducible microreactors to discretize reagents into picoliter or nanoliter volumes [1,2]. Benefiting from isolated microenvironments, precise manipulation of the number of cells inside each droplet makes it possible to study phenotypic and genetic heterogeneity at the single-cell level. Accordingly, it is essential to generate and monitor highly uniform droplets with single-cell encapsulation for single-cell analysis [3,4]. 

Several methods have been proposed for the passive generation of uniform microdroplets [5]. The coflowing structure was first introduced while the dispersed phase and continuous phase coaxially flowed along the inner and outer coaxial capillaries, respectively [6]. The dispersed phase with cell contents formed a flow focusing inside the continuous phase and automatically broke into droplets with encapsulated cells due to capillary instability. Correspondingly, flow-focusing structures were also applied where the dispersed phase received the combined force of the pressure drop and shear stress exerted by the continuous phase [7]. It has shown a good advantage in throughput since smaller droplets were periodically generated at a higher frequency. Similarly, in the T-junction structure [8], the dispersed phase is perpendicular to the continuous phase and changes the symmetric force into an asymmetric force. In addition, the interface curvature of the two phases could suddenly decrease within an abrupt change in the channel dimension, resulting in a decrease in the Laplacian pressure and producing outward drag [9]. Considering their reproducibility and robustness, flow-focusing microfluidic chips fabricated by soft lithography processing have been widely used to generate homogeneous microdroplets for cell encapsulation.

With limited exceptions, the number of cells encapsulated per droplet using passive methods is restricted by Poisson statistics [10], where a theoretical maximum of 36.78% (1/e) of all droplets contain exactly one cell. However, even at the cost of specificity, 26.42% of droplets will contain at least two cells, namely, the covalence probability, reducing the effective rate of single-cell encapsulation. Recently, many efforts have been made to break the inherent limits by regularly ordering cells in inertial [11,12] or close-packed channels, on-demand encapsulation, and post-encapsulation sorting, while massive microdroplets are produced with a generation rate on the order of 1~10 kHz. Instead of manual cell counting and analysis, this growing advance has created an active need for computational tools capable of processing many droplets. In addition, various parameters related to the microdroplet formation process will affect the final size distribution and encapsulation rate and need to be periodically monitored with quality control [13]. Therefore, it is highly necessary to develop complementary and automatic methods to evaluate the equality of generated droplets and quantify the encapsulated contents.

There has been some research [14,15,16,17,18] focusing on these tasks based on the video frames acquired by a high-speed camera in which the droplets are clearly separated. This dynamic strategy suffers from fuzzy motion and neglects droplet fusion that occurs downstream of the imaging scope. Hence, the monitoring of dynamic droplet generation is useful but insufficient. A two-stage object detection framework [19,20,21] has been applied to static microscopic images to separately recognize the generated droplets and the encapsulated cells. In the first stage, available droplet proposals are generated with their boundaries segmented by masks. Considering the differences between droplets and oil backgrounds, morphological analysis is first applied to yield droplet proposals. Some research extracts their edge feature maps and adopts the Hough transformation to find circular-like contours [15,22,23]. Background models and connected component analysis [16,24] are applied to segment the droplet foreground and locate droplet proposals. These methods work appropriately on transparent and separate droplets while encountering difficulties with opaque and adherent droplets. In the second stage, there are two kinds of approaches for detecting encapsulated contents and classifying droplets, including morphological analysis and machine learning algorithms. Basu et al. [14] directly judged whether droplets encapsulated any particles by quantifying the deviation of their internal grayscale. Similarly, the standard deviation in the distance between the contour and gravity center of droplets is employed [19]. However, these morphological methods rely heavily on imaging quality and cannot count the cell number. Adopting machine learning, the random forest was utilized to precisely detect beads inside each droplet with manual labeling [20]. Handcrafted features were fed into a support vector machine (SVM) to classify droplets into empty, single-cell, and multicell encapsulations [25]. Convolutional neural networks (CNNs) were also employed [26] to classify encapsulated droplets. Although these methods can extract superficial droplet or cellular information, they are frequently restrained in their ability to quantify cell encapsulation and require considerable model parameters and excessive training time.

Reconsidering the recognition task of cell-encapsulated droplets, we realized that the key difference is the cell quantity rather than the divergence of cell-like features. Traditional classifiers can generally learn the bias among different categories; unfortunately, they cannot count the cell population in each droplet. Research on cell counting mostly adopts regression approaches to estimate the density map of a given medical image [27], while the integral of the density map might intuitively indicate the cell number. These fully supervised learning approaches require tedious cell-level annotation for the training procedure, including a precise cell population [28] and the accurate location of each cell [29]. To avoid time-consuming annotation, three droplet-level labels (empty, single-cell, and multicell encapsulation) instead of cell-level labels are adopted in this paper. Accordingly, we developed a microfluidic system embedded with a recognition algorithm to generate microfluidic droplets, monitor droplet size, and further recognize the encapsulated numbers of cells. A morphological approach named adaptive scale template matching (ASTM) was first proposed to generate proposals. Second, to distinguish and categorize droplets by the number of encapsulated cells, the cell population inside each droplet was estimated by a weakly supervised cell counting network (WSCNet). Next, this algorithm also provides the location prediction of each cell, which is more explainable and reasonable compared with a CNN-based classifier. In addition, we verified our approach with intricate droplet data collected from three different microfluidic structures under different experimental parameters. Quantitative experimental results showed that our approach can not only distinguish droplet encapsulations (F1 score > 0.88) but also locate each cell without any supervised location information (accuracy > 89%). We also demonstrated the feasibility of this combined microfluidic system and cell counting network approach towards single-cell encapsulation analysis. The probability of “single cell in one droplet” encapsulation is systematically verified under different parameters and is in good agreement with the Poisson distribution. The whole system is self-contained, and the proposed counting networks are tiny and effective, which can be easily employed as a comprehensive platform for the quantitative assessment of encapsulated microfluidic droplets.

## 2. Working Principle

### 2.1. Principles of Droplet Generation

**Figure 1:**
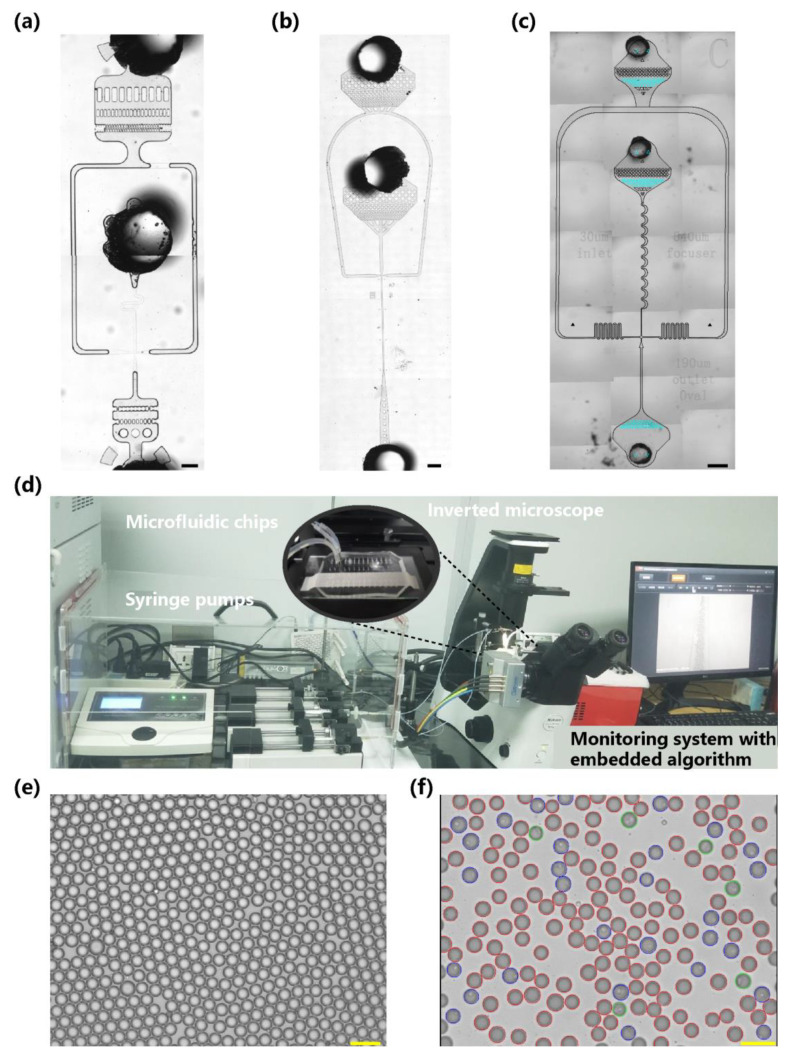
A microfluidic system embedded with a recognition algorithm for the generation and recognition of cell-encapsulated droplets. (**a**–**c**) Optical micrographs depicting the geometry of the A-type, B-type, and C-type microfluidic chips for cell encapsulation. (**d**) Photograph of the droplet generation system composed of a microfluidic chip, multichannel syringe pumps, an inverted microscope, a USB camera, and a computer embedded with our proposed algorithm. (**e**) Representative initial microscope image of the generated droplets with cell per droplet (CPD) *λ* = 0. (**f**) Representative result of droplet segmentation and cell classification with CPD *λ* = 0.22. The droplets were divided into empty, single-cell, and multicell encapsulations (red, blue, and green circular masks, respectively) according to the encapsulated cell count. The scale bar represents 100 μm.

The flow-focusing structures were characterized by square cross sections of identical height *h* that intersected at right angles, as shown in Figure 1a,b. Two continuous phase channels symmetrically intersected with a dispersed phase channel on both sides and exited from the outlet channel. Different flow regimes were observed in the flow-focusing channel, including squeezing, dipping, jetting, and tubing regimes [10,30]. In addition, several submodes can also be observed depending on the details of the chip design and the flow rates of the dispersed and continuous phases [31]. This study focuses on the jetting mode and its transition from the dripping mode for cell encapsulation. Supplementary Figure S1 shows representative pictures of these regimes.

Although several numerical simulations have been reported [32], there is no fully defined theoretical model that can quantitatively explain the relevant parameters and critical points of all regimes [33]. It is qualitatively explained that droplet formation in the dripping and jetting regimes is affected by end-pinching and Plateau-Rayleigh instability, respectively [34]. Rayleigh instability confers surface tension and magnifies the natural disturbance in the fluid thread, while the end-pinching mechanism is due to the pressure gradient of the continuous phase squeezing the neck of the fluid thread [35]. The transition from dripping to jetting regimes can also be regarded as the Rayleigh instability changing from local to convective regions [31]. 

### 2.2. Droplet Encapsulation and Poisson Distribution

Diluting cells into the dispersed phase before droplet formation is one of the most common methods for single-cell droplet encapsulation. The basic principle is to adequately dilute the cell suspension (*λ* < 1), ensuring that no two cells appear inside the same droplet. When the droplet is generated in a uniform and stable manner, the probability distribution of the cell number in the droplet follows the Poisson distribution [10]. Assume the average number of cells in each microdroplet (cell per droplet, CPD, cell density divided by droplet volume) is *λ*; then, the probability of *k* cells in one microdroplet can be calculated according to:

(1) $$\rho \left(k\right)=\frac{\lambda^{k}}{k!}exp\left(-\lambda \right)$$

Supplementary Figure S2 and Note S1 show that the single-encapsulated, multicell-encapsulated, and encapsulated rates increased to different degrees, while the single-cell rate (the proportion of droplets containing exactly one cell to those containing cells) decreased rapidly with increasing *λ*. Considering the results of cell detection in the droplet, the proportion of positive droplets is:

(2) $$\frac{M^{d}}{N^{d}}=1-\rho \left(0\right)$$

where *N^d^* and *M^d^* are the numbers of detected microdroplets and positive microdroplets, respectively. According to Equation (1), the observed value *λ* of CPD can be calculated as follows:

(3) $$\lambda =-ln\left(1-\frac{M^{d}}{N^{d}}\right)$$

Therefore, the posterior *λ* depends on three elements: uniformity of the formation process of microdroplets (ensuring the cell number in a droplet meets the theoretical distribution), detected microdroplets *N^d^*, and positive microdroplets *M^d^*. The first element is related to the microdroplet formation process, and the last two elements are related to the sampling process.

### 2.3. Droplet Recognition and Cell Counting

**Figure 2:**
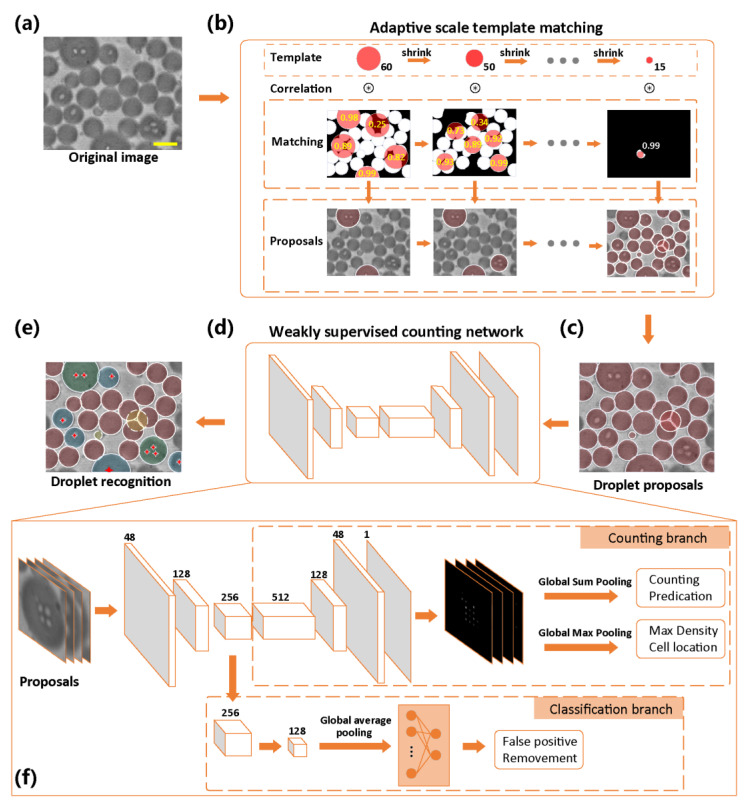
The framework of our proposed approach. (**a**) The original input for the framework is a full-frame image with highly adherent droplets. (**b**) Details of the adaptive scale template matching (ASTM) algorithm. (**c**) Droplet proposals are first generated by ASTM to segment all droplets. (**d**) Proposals are then fed to the weakly supervised counting network (WSCNet), which not only removes false positive proposals but also locates all cells in true positive droplets. ASTM and the WSCNet are highlighted with an orange block diagram, while their corresponding functions are droplet proposal generation and cell recognition, respectively. (**e**) False positive proposals and cells are marked by a yellow circular mask and labeled by red points, respectively. According to the number of encapsulated cells, all droplets are reclassified into empty, single-cell, and multicell encapsulations, marked by red, blue, and green circular masks, respectively. (**f**) The detailed architecture of the WSCNet consists of classification and counting branches, which perform cell counting and localization or act as filters to remove false positive proposals, respectively. The scale bar represents 50 μm.

To realize the objective statistics of the droplet quality and the number of encapsulated cells, we developed an automatic algorithm for the image recognition of cell-encapsulated droplets. Figure 2 exhibits the whole procedure of our approach, in which droplet proposals are first generated by adopting ASTM, and second, the proposals are fed to a WSCNet to remove false positive proposals and further count the cell population of true positives.

For small target segmentation of dense and adhesive droplets, ASTM is proposed by using adaptive shrinkage according to the response of circular template matching and foreground image binarization. Next, a greedy search is utilized to locate the candidate droplets, as shown in Figure 2b, and then the non-maximum suppression filter is used to remove the redundant concentric circles and generate the final segmentation of droplet proposals. Finally, to realize cell counting and localization in droplets, we proposed a WSCNet based on a weakly supervised CNN. The training process of the model only needs droplet-level labels, providing the droplets with two kinds of precise numbers (sterile, one) or one fuzzy number (at least two). We optimized the truncation loss function according to the label and the weak supervision strategy, which in turn accurately predicted the cell counts and location by the integral and the maximum of the density maps.

## 3. Materials and Methods

### 3.1. Microfluidic Chips and Experimental Platform

Hydrodynamic flow-focusing structures were used to generate droplets, while bacterial cells were successively encapsulated. We constructed a microfluidic droplet generation platform, as shown in Figure 1d, including different microfluidic chips, a multichannel syringe pump (TS-1B, Longer Precision Pump Co. Ltd., Baoding, China), an inverted microscope (Motic AE31, Panthera, Xiamen, China), a USB camera (acA1920, Basler Asia Pte. Ltd., Singapore) with a resolution of 1920:1200, and a computer embedded with our proposed algorithm. The camera and microscope with 10/20/40× objectives provided clear images of cells larger than 2 μm. Three microfluidic chips with different geometries were established, as shown in Figure 1a–c, to compare and validate the generalization ability of the algorithm on intricate data collected from different chips. The first two geometries generated the encapsulated droplets based on passive methods, while a serpentine inertial focusing channel [11] was added to the third geometry to preorder cells and attempt to improve the single-cell encapsulation rate.

All microfluidic layers were fabricated using standard soft photolithography with patterns etched on silicon wafers [1]. Master molds with 20–40 μm-thick SU-8 were fabricated in a clean room. The PDMS (polydimethylsiloxane) base and its curing reagent (Slygard 184, Dow Corning, Midland, MI, USA) were thoroughly mixed and degassed in a vacuum oven. Next, the PDMS mixture was cast onto the SU-8 molds, cured at 85 °C for 1 h, and peeled off from the molds. The PDMS slab was cut into a suitable size, punched for inlets and outlets, and bonded to a glass substrate after oxygen plasma treatment (Femto, Diener Electronic, Ebhausen, Germany). Aquapel (PPG Industries, Pittsburgh, PA, USA) was injected into the microchannels and blown out after 5 min for surface modification.

The encapsulated droplets were captured at two periods for training and inference purposes. The first period lasted for two months, and 830 images with a resolution of 640 × 480 were collected with a mean of 191.55 droplets per image for algorithm training. All the images were randomly divided into a training set, a validation set, and a test set according to a ratio of 4:1:1. There were four types of samples to be classified: background, empty, single, and multiple, while the background samples were extracted by random sampling on the background of the droplet images. Only three droplet-level labels were provided for the training stage. To quantify the localization performance of our approach, we pinpointed the center of each cell (diameter 3–10 μm) in the test dataset of multicell encapsulation, which was not applied in the training procedure. The number of samples in each dataset is shown in Table 1. 

Since the population of empty droplets is far larger than the population of single and multiple encapsulations, a random sampling subset of the empty samples was adopted in the training, validation, and testing. The number of background samples was approximately equal to the sum population of the other three samples, while the former and the latter were used as the negative and positive samples for the binary classification branch, respectively.

The second period followed the first period and lasted for six months; its purpose was to verify the generalization performance of the proposed algorithm on multitasking. Ninety-three groups of cell-encapsulated droplet generation are implemented for inference, each under different parameters, including microchip structures (A/B/C type), flow rates of the dispersed and continuous phases (Q_1_ and Q_2_), and CPD (*λ*), to generate different image distributions. More than 1800 images were collected from three microfluidic geometries (more than five chips were tested for each geometry), each containing 100~250 droplets.

### 3.2. Convolutional Neural Network-Based Imaging Recognition

We proposed a CNN-based recognition algorithm to evaluate droplet quality (size and distribution) and further recognize the encapsulated cell (amounts and position) in two stages. In the first stage, ASTM is proposed to heuristically generate droplet proposals from the binary foreground of highly adherent droplet images segmented by the Otsu algorithm, as shown in Figure 2b. Specifically, assuming a circular binary template is denoted by T and its initial diameter is *r*, the matching response in the foreground *D* (*x*, *y*) can be computed by:

(4) $$D\left(x,\;y\right)=\sum_{a,b\;\in \;\left[0,r\right)}T\left(a,b\right)F\left(x+a-\frac{r}{2},y+b-\frac{r}{2}\right)$$

where F denotes the binary foreground image. *D* (*x*, *y*) is essentially the ratio of the foreground area inside the template to the full template area. Consequently, its maximum corresponds to the largest foreground area covered by the template, which can be computed by $\pi r^{2}D_{\max}$. Droplet proposals can be generated by the greedy search of all local maxima. 

Nevertheless, the diameter of the droplets varied due to unpredicted fusion or flow disturbances. A template with a minor *r* can locate small droplets but yield an inaccurate $D_{\max}$ for large droplets, and vice versa. It is necessary to elaborate an adaptive scale template to find all droplets with different diameters. The pixels with $D_{\max}$ higher than a predefined threshold *σ* were marked as the center of a droplet proposal, while the current template scale suggests its diameter. In contrast, if all $D_{\max}$ are less than *σ*, the next template scale is adaptively shrunk by:

(5) $$r_{next}=\sqrt{D_{\max}\times r_{cur}}$$

Since overlapping bounding circles might occur in one true droplet, non-maximum suppression (NMS) [36] is employed to remove redundant circles, as shown in Supplementary Figure S3. 

In the second stage, the cell-encapsulated droplets were detected using droplet proposals. We developed the WSCNet to estimate the number of cells and predict their positions. To avoid tedious and manual cell-level annotation, only three droplet-level labels, including empty, single-cell, and multicell encapsulation (0, 1, >1), are adopted. The WSCNet consists of classification and counting branches, as shown in Figure 2f. The former serves as a filter to remove false positives from previously generated proposals. Similar to other counting tasks [27,28], the output of the latter branch is a single-channel density map, and its integral and local maxima may indicate the number and location of cells, respectively. Cross entropy was adopted by the classification branch as the loss function $L_{class}$ to provide a predicted label (droplet or false positive). The counting branch may employ the mean square error (MSE) between the label and the prediction as a loss function:

(6) $$L_{count}=\|f\left(D\right)-y{\|}^{2}$$

where *y* suggests the supervision, i.e., the true counting label, and D and $f\left(D\right)$ denote the output density map and its counting prediction, respectively. Considering that the multicell encapsulation contains at least 2 cells, we quantify its label as 2 and truncate its counting prediction to 2, which can be formulated by:

(7) $$f\left(D\right)=\left\{\begin{array}{c} D_{sum}\;\;\;\;\;\;\;\;\;\;D_{sum}<2\\ 2+\gamma \times D_{sum}\;\;\;\;\;\;D_{sum}\geq 2\;\;\;\;\;\;\;\;\;\; \end{array}\right.$$

where $D_{sum}=\underset{i,j}{\sum }D\left(i,j\right)$ indicates the integral of the density map obtained by global sum pooling and *γ* represents a small constant that provides a gradient. A regularization performed on each density value is added to Equation (6) to avoid overestimating the counting prediction caused by the truncation:

(8) $$\psi \left(D\right)=\left\{\begin{array}{c} D_{max}-1\;\;\;\;\;\;D_{max}>1\\ \;\;\;\;\;\;\;\;\;\;\;0\;\;\;\;\;\;\;\;\;\;\;\;\;\;\;\;D_{sum}\leq 1\;\;\;\;\;\;\;\;\; \end{array}\right.$$

where $D_{max}$ represents the max value in the density map obtained by global max pooling. The loss function Equation (6) for the counting branch can be rewritten as follows:

(9) $$L_{count}=\|f\left(D\right)-y{\|}^{2}+\psi \left(D\right)$$

Finally, the loss function of the whole network containing two branches is given with a weight of *ω*:

(10) $$L=\omega \times L_{count}+\left(1-\omega \right)\times L_{count}$$

The classification branch provides a predicted label, and the counting branch outputs a density map with the same resolution as the input image. The density map is valid only when the predicted label is a droplet. $D_{sum}$ suggests the number of encapsulated cells, and the first $\left\lfloor D_{sum}+0.5\right\rfloor$ maxima in the density map indicate the cell location. According to the predicted cell numbers, it is easy to reclassify the droplets into three categories (empty, single cell, and multicell) for comparison with other classification-based approaches:

(11) $$id=\arg\underset{k\;\in \;\left\{0,\;\;1,2\right\}}{\min}{\left(D_{sum}-k\right)}^{2}$$

More details on the proposed algorithm are described in Supplementary Notes S2 and S3.

### 3.3. Network Implementation and Evaluation Metrics

We set the matching response threshold *σ*, the small constant *γ*, and the weight *ω* to 0.98, 0.001, and 1, respectively. ReLU is adopted as the activation function in the whole network. The batch size is set to 1024. The learning rate is initialized at 10^−4^ and adjusted by the loss of the validation set. Inspired by the interaction over union (IoU) of bounding boxes in object detection issues, we adopt a bounding circle IoU to distinguish the true positive predictions, as shown in Figure S4, which is calculated by:

(12) $$\mathrm{IoU}=\frac{Area\left(C_{gt\;}\cap \;C_{pre}\right)}{Area\left(C_{gt}\;\cup \;C_{pre}\right)}$$

where *C_gt_* and *C_pre_* represent the true and predicted bounding circles of a droplet, respectively. A droplet proposal with an IoU higher than a threshold *θ* indicates a true positive prediction.

We evaluate our algorithm from three angles. First, recall and precision are adopted to evaluate the performance of the ASTM on the generation of droplet proposals. Second, the metric F1, computed by Equation (13), is employed to quantify the performance of the WSCNet on the recognition of the encapsulated cell number. Additionally, the F1 score, model size, and training time are utilized to compare with other classification-based approaches. Third, to quantify the location performance of our approach, a circular mask with an *x*-pixel radius centered at each annotated cell centroid is regarded as a valid area. Accordingly, a location prediction that is nearest to a cell centroid and falls into its valid area is a true positive; the metrics are also adopted to evaluate the location performance of the WSCNet.

(13) $$F1=\frac{2\times Precision\times Recall}{Precision+Recall}$$

In addition, since our algorithm can provide the exact cell population of each droplet, the cell number in the droplets can be constructed. We, therefore, selectively labeled the exact cell population of the multicell encapsulation to evaluate the predicted number, which is not applied in the training procedure. The counting performance can be measured by the mean relative error (MRE).

### 3.4. Experimental Setup for Droplet Generation and Cell Encapsulation

The microfluidic chips were placed on the stage of an inverted microscope for observation and recording. Multichannel syringe pumps were used to inject the dispersed and continuous phases into the corresponding inlets. In most generation experiments, a mixture of mineral oil was used as the continuous phase, including 3% (*w*/*w*) EM90 (ABIL, Evonik, Essen, Germany), which served as a surfactant, decreasing the surface tension, and 0.1% (*v*/*v*) Triton-100 dissolved in light mineral oil (M5310, Sigma–Aldrich, St. Louis, MO, USA). In partial experiments, Novec 7500 (3 M Inc., St. Paul, MN, USA) with 1% dSURF surfactant (DR-RE-SU, Fluigent, Le Kremlin-Bicêtre, France) was used as the continuous phase.

Yeast cell solution in PBS premix was used as the dispersed phase, consisting of 40% (*v*/*v*) OptiPrep medium (D1556, Sigma–Aldrich) or 30~50% glycerol (356350, Sigma–Aldrich) and PBS to prevent cell sedimentation. Yeast (*Saccharomyces cerevisiae*) was cultured for standard resuscitation at 28 °C in a YPD medium (formulated with 20 g of glucose, 10 g of yeast extract, and 10 g of peptone dissolved in 1 L of distilled water). After well blending, the diluted cells were incubated at room temperature, and the cell fractions were washed twice by resuspending in PBS and discarding the supernatant after 1000 rpm centrifugation for 5 min to remove residual debris and doublets. Before sample mixing, 10 µL of cell solution was stained for activity analysis and density calculation (*D*_cell_).

In all experiments, droplets were generated within the microfluidic chips by injecting the dispersed and continuous phases at designated flow rates. The cell-encapsulated droplets were collected into EP tubes, and one drop was added onto glass slides pasted with rectangular enclosures that were prepacked with oil. Microscopic images of droplets were acquired with a USB camera, while the mean size (*R*_cell_) and coefficient of variation (CV) were automatically calculated with the proposed algorithm. Therefore, the average number of cells in each microdroplet (CPD, *λ*) was quantified as the cell density *D*_cell_ divided by the droplet volume 4/3π*R^3^*_cell_. The cell counts of the encapsulated droplets were automatically analyzed and labeled by the proposed method. Multiple statistical results were fitted to the Poisson distributions, with error analysis conducted by the Chi-Squared and Residual Sum of Squares (RSS) tests.

## 4. Results and Discussion

### 4.1. Preliminary Experimental Analysis of Droplet Generation

**Figure 3:**
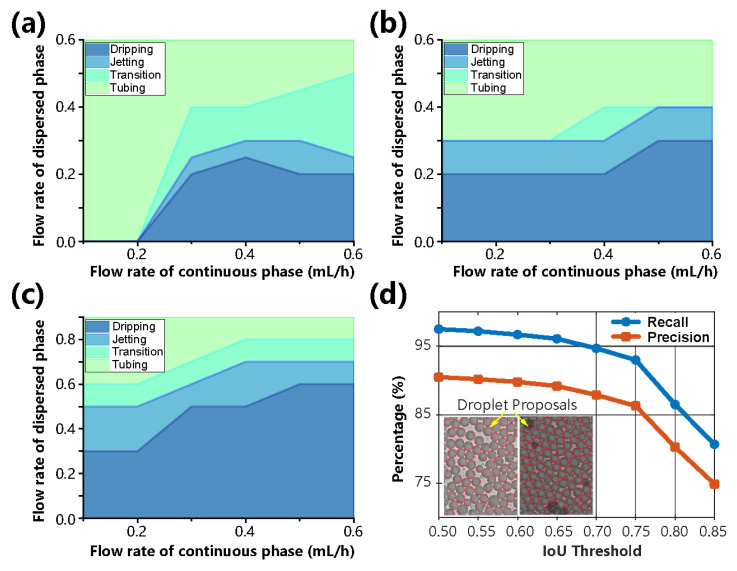
Mode diagrams of droplet generation as a function of the two-phase velocity. (**a**) Flow patterns of a 30% glycerol-PBS solution as the dispersant phase and a surfactant-added mineral oil solution as the continuous phase. (**b**) Flow patterns of a 30% glycerol-PBS solution as the dispersant phase and mineral oil as the continuous phase. (**c**) Flow patterns of a 50% glycerol-PBS solution as the dispersant phase and mineral oil as the continuous phase. The flow rates of the dispersed and continuous phases are introduced as Q_1_ and Q_2_, respectively. *n* = 3 independent experiments. (**d**) The performance results of recall and precision at different IoU thresholds in recognizing droplet proposals. Inset: predicted droplet proposals marked with red circles.

Preliminary experiments with the droplet system are crucial for the transition from recorded images to recognized data, as mentioned in the working principle and Equation (3). The uniformity in the droplet formation process is essential for cell encapsulation, which is highly influenced by flow regimes; e.g., droplets are not stably generated in the tubing or transition regime. The relationships of droplet generation between different regimes are summarized as flow patterns according to the flow rates of Q_1_ and Q_2_. As shown in Figure 3a–c and Figure S1 when Q_2_ was fixed and Q_1_ increased, the flow transitioned between dripping, jetting, and tubing regimes. However, the reverse transition occurred in most cases when Q_1_ was fixed and Q_2_ increased. This trend is intuitively understandable: an increase in Q_1_ means that its occupied area at the exit of the cross channel will be larger, so it will take a longer distance for the disturbance to expand until the dispersed phase breaks into droplets. In addition, the increase in the total flow rate allows the fluid to move a longer distance at the same interval. Growth in Q_2_ also had a stronger effect on reducing the cross-sectional area of the dispersed phase. Therefore, the transition direction was usually from the tubing to the jetting and then to the dripping regime.

From the comparative study, it is not difficult to conclude that at the same Q_2_ flow rate, reducing the surface tension (surfactant) and increasing the viscosity of the dispersed phase (glycerol ratio) can make the regime change from dripping to jetting at a lower Q_1_ flow rate. This is a logical result when the transition from dripping to jetting is considered a transition in Rayleigh’s theory from local instability to convective instability. Similarly, decreasing the surface tension and increasing the viscosity both moved the jetting-tubing boundary down. In addition, we also found the capillary numbers C_Q1_ and C_Q2_ to be more relevant in terms of the influence of physicochemical properties. According to the independent change in Q_1_ or Q_2_ over at least one order of magnitude, it is suggested that the transitions between dripping and jetting usually occur around the critical value C_a*c*_ ≈ 10^−1^, which is consistent with a previous study [31]. These results were reproducible for different batches of microfluidic chips, as shown in Figure 1e,f.

### 4.2. Quantitative Performance of Droplet Recognition

**Figure 4:**
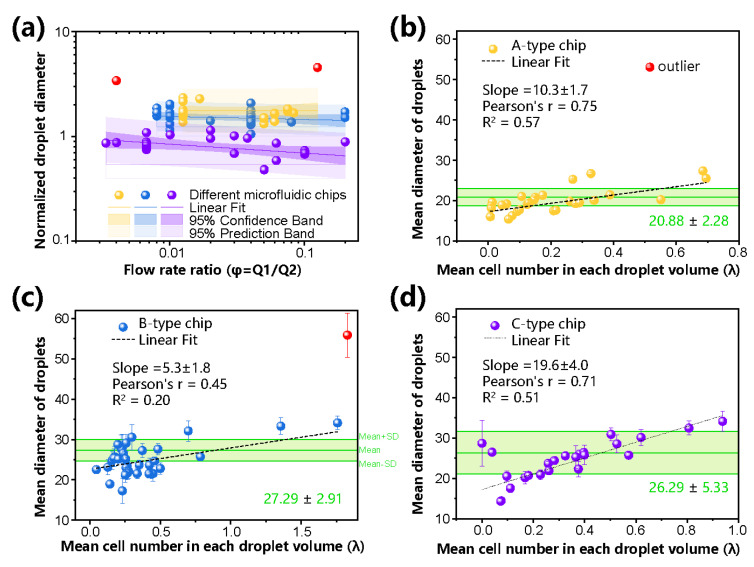
The size uniformity of generated droplets can be characterized and monitored with the proposed method. (**a**) Relationship between normalized jetting droplet diameter (droplet diameter divided by the length scale of the nozzle) and the flow rate ratio *φ* (Q_1_/Q_2_). (**b**–**d**) The mean diameters of generated droplets were calculated under different mean numbers of CPD *λ* on A-type, B-type, and C-type microfluidic chips. The mean diameters of the generated droplets are 20.88 ± 2.28, 27.29 ± 2.91, and 26.29 ± 5.33 μm. More than 1500 droplets are measured in each independent experiment. Chip symbol: yellow A-type, blue B-type, and violet C-type microchips.

To validate the sensitivity of droplet proposals generated by the ASTM approach, we evaluated its performance under different IoU thresholds *θ*, from 0.5 to 0.85 with a step of 0.05, as shown in Figure 3d. In the case of object segmentation, IoU evaluated the overlap between the ground truth and the prediction region. When *θ* was set to the traditional 0.5, the recall and precision were greater than 0.97 and 0.9, respectively, which suggests that the droplet proposals cover more than 97% of the true droplets and more than 90% of the droplet proposals are true positives. In addition, the recall was still greater than 0.93 even though *θ* rose to 0.8 (a tougher condition), indicating that ASTM is robust and insensitive to the thresholds *θ*. Insets of Figure 3d and Figure S5 also show representative results of droplet proposals in some special images, including multiscale and highly adherent droplets. In addition, the ASTM algorithm also calculated the droplet diameter and distribution deviation, as shown in Figure 4 and Supplementary Video S1.

To evaluate the droplet generation under different parameters, we recorded and recognized the static images of generated droplets on A/B/C chips at different flow rates of Q_1_ and Q_2_ and CFD *λ*. For example, using the A-type chip, the same compatibility ratio of dispersed and continuous phases, and different *λ*, we set the flow rate of the dispersed phase to 0.06 mL/h and adjusted the Q_2_ flow rate in the range of 0.8 to 1.2 mL/h. The droplets were obtained with a mean diameter of 19 μm and a CV of less than 4% and verified by repeated experiments (*n* > 5). The droplet was also generated in the B-type chips at a frequency of 6 kHz, and the droplet size CV was approximately 2.6%, indicating good monodispersity. Considering the independent change in *φ* = Q_1_/Q_2_ over at least one order of magnitude, as shown in Figure 4a, the droplets generated by A-type and B-type chips shared similar normalized droplet diameters (diameter divided by the length scale of the nozzle) of 1.76 ± 0.14 and 1.50 ± 0.06, respectively. In contrast, the droplets generated by C-type chips showed less normalized droplet diameter, which also decreased with increasing flow rate ratio *φ*. To compare the influence of encapsulated cells on the diameter of droplets, we calculated the average diameter and standard deviation at different *λ* ratios, as shown in Figure 4b–d. More than 1500 droplets are measured in each independent experiment. The mean diameters of the generated droplets on different microchips were 20.88 ± 2.28, 27.29 ± 2.91, and 26.29 ± 5.33. In addition, it was obvious that as the CFD *λ* increased, the cell burden on droplets increased, so that the droplet diameter increased in response. These experiments also showed that in the C-type chip, the diameter is most susceptible to the influence of *λ*, as the slope (19.6 ± 4.0) of the fitting curve was significantly higher than that of the other groups (*p* value < 0.01). In contrast, the B-type chip with a straight inlet channel demonstrated a more independent diameter. These results suggested differentiated applications for the different chips, while the performance of the inertial focusing channel depended on the flow rates and CFD *λ*.

### 4.3. Quantitative Performance and Comparison of Cell Recognition

**Figure 5:**
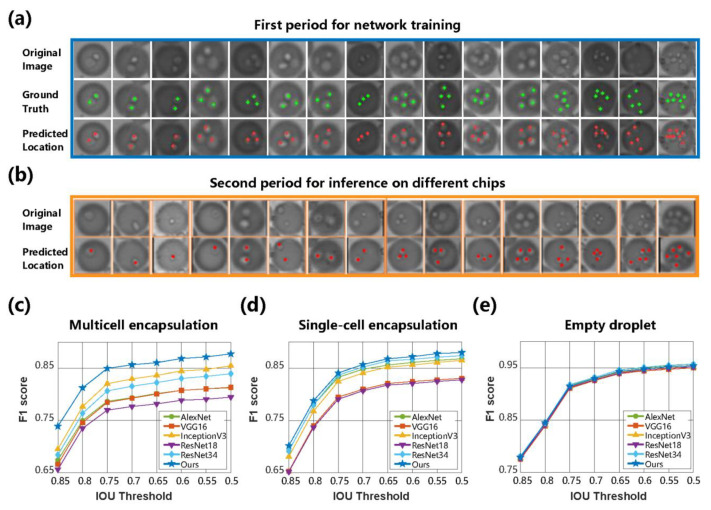
Comparative results of image recognition algorithms for cell-encapsulated droplets. (**a**) Visualization of cell location in the first period for network training and testing. Only three droplet-level labels were provided for this training stage. The ground truth of each cell in the test dataset of multicell encapsulation is marked as green points, which were not applied in the training procedure. (**b**) Visualization of cell location in the second period for inference on three different microfluidic chips. The first rows denote the original droplet images. Red points mark the predicted location of each cell. (**c**–**e**) The F1 values of encapsulation recognition on each droplet type, including multicell, single-cell, and empty droplets, were derived from comparative methods under different IoU thresholds.

For the cell recognition stage, we quantified the cell location performance under different radii ranging from 4 to 10 pixels, which is shown in Supplementary Figure S6. To quantify the localization performance, we first pinpointed the center of each cell in the test dataset of multicell encapsulation, which was not applied in the training procedure. According to the location of the ground truth pixel and the predicted pixel by our method, a circular mask with a preset threshold of the x-pixel radius centered at the annotated cell centroid was regarded as a valid area. It can be assumed that more than 80% of cells in droplets were precisely located when the radius was set to 10 pixels; meanwhile, over 89% of location predictions fell into valid cell areas. Even though the radius drops to 5 pixels, the precision and recall remain above 0.73 and 0.66, respectively, indicating that the proposed WSCNet effectively achieved the assignment of cell location. Figure 5a shows representative results of cell location in droplets, with green and red points marking the ground truth and predicted location, respectively. The encapsulated cells were detected and localized from the droplet proposals in the second period for inference on different chips, as shown in Figure 5b. Reconsidering the weak supervision of droplet-level labels, it can be explained that the network learns both cell features and cell numbers from the difference between empty and single-cell encapsulation and identifies the learned features to determine multicell droplet encapsulations.

Classification-based algorithms were applied as benchmarks by classifying the proposals into different categories. Because these algorithms only categorize the droplet proposals into three categories, Equation (11) was adopted to reclassify the cell counting result of our algorithm for the comparative study of the entire process. The metric F1 score was adopted to quantify the overall recognition performance of the three droplet categories under different *θ* values ranging from 0.5 to 0.85. Comparing the WSCNet with AlexNet [37], VGG16, Inception V3 [38], ResNet18, ResNet34 [39], and other classification networks, it was found in Figure 5c–e that the WSCNet algorithm showed the highest F1 scores in empty, single-cell, and multicell encapsulated droplets. The increase in *θ* indicated a tougher judgment criterion for true positives and consequently led to a decrease in the F1 score of each category. It is noticeable that the F1 scores did not drop significantly until *θ* rose to 0.8, suggesting the same conclusion as the performance quantification in the droplet proposal generation stage.

At the junction ratio of *θ* = 0.7, the F1 scores of the WSCNet were 0.86, 0.86, and 0.93, showing a mean F1 score > 0.88. In addition, our approach counted the number of cells without the heavy burden of manual annotation on the precise number (only 0, 1, or > 1 was provided). Therefore, it outperformed other classification-based algorithms by a large margin in recognizing multicell droplet encapsulations. It is important to note that the WSCNet not only counted the cell population but also located cells without any location information in the network training by searching the local maxima in the density map. Some visualization examples are shown in Figure 5a,b, in which the counting network managed to locate cells with different scales in droplets.

Table S1 summarizes the recognition performance in the comparative study. It is obvious that most of the approaches achieved comparable performance in recognizing empty droplets, while our counting-based method ranked first in distinguishing both single-cell and multicell droplet encapsulation. Our method achieved nearly the same recognition performance as ResNet34 and maintained only one-eighth the size of the model. Although VGG16 and InceptionV3 achieved better performance on the recognition of empty encapsulated droplets, their model size was much larger, and their training time was nearly ten times that of ours. In addition, the model parameters of the proposed network occupied the smallest memory, suggesting that our algorithm is small and effective. More than 300,000 images of droplets are verified by the proposed algorithm, and the MRE of the cell count was 5.96% for droplets with single cells and 12.54% for droplets with multiple cells. It is worth noting that this weakly supervised learning strategy avoids pixel-level labeling of cell location.

### 4.4. Independent Test Performance on Cell-Encapsulated Droplets

We further verified this microfluidic droplet platform embedded with the proposed algorithm for multitask recognition performance on cell-encapsulated droplets. The images of cell-encapsulated droplets were further analyzed after generation from microfluidic chips with different geometries at different *λ*. It has been proven that droplets with a size of 14–26 μm can be stably generated with a diameter deviation of less than 4% at a generation frequency of 2–9 kHz. Typical values of single-cell and multicell encapsulation rates were 24.89% and 3.74%, 17.75% and 1.63%, and 24.05% and 3.72%, respectively, given by the recognition algorithm. In addition, the representative result of the cell number distribution is exhibited in Supplementary Figure S7 with CPD λ = 0.16 encapsulated in microfluidic droplets. The single-cell and double-cell encapsulation rates were approximately 12% and 2%, respectively, while less than 1% of the total drops encapsulated more than two cells. In this inference stage, it is suggested that there is no significant difference (*p* value < 0.05) between the number predicted by the proposed method and the true cell count.

**Figure 6:**
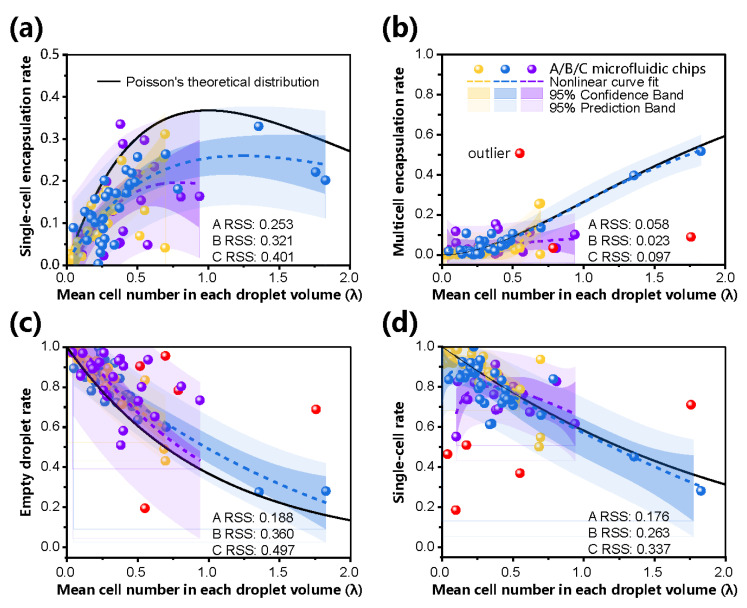
Evaluation results of cell-encapsulated droplets generated at different parameters. Plots of the experimentally measured, nonlinear fitting, and Poisson’s theoretical distribution of the single-cell encapsulation rate (**a**), multicell encapsulation rate (**b**), empty droplet rate (**c**), and single-cell rate (**d**). The results were systematically verified under different values of *λ* on three different chip geometries. Each data point represents different flow rates (Q_1_ and Q_2_) or CPD *λ*. More than 1500 droplets were measured in each independent experiment. Chip symbol: yellow A-type, blue B-type, and violet C-type microchips.

Finally, we summarized the recognition results of 93 group images of cell-encapsulated droplets under different parameters. The comparison between the experimental results, their nonlinear fitting, and the theoretical distribution of different droplets is plotted in Figure 6 under different CFDs *λ*. It was first concluded that the overall variation tendency, including the empty, single-cell, and multicell encapsulation rates and the single-cell rate, was basically consistent with Poisson’s distribution curve because all their RSS tests were less than 0.5. Especially for the multicell encapsulation rate in Figure 6b, the experimental results of A-type and B-type chips are in accord with Poisson’s distribution with an RSS < 0.06. In addition, as shown in Supplementary Note S4, different nonlinear curves were applied for the approximated fitting of the theoretical distributions on the encapsulation rates and the single-cell rate. From the graph in Figure 6, it was noticed that the 95% prediction band of nearly all groups covered the related theoretical curves. The relatively wide prediction band may be caused by a deviation in the droplet diameter, which is cubically utilized for calculating CFD *λ*.

In addition, except for the single-cell encapsulation rate, the 95% confidence band of most groups covered or was close to their related theoretical curves. As shown in Figure 6a, the nonlinear fitting curves of all three microfluidic geometries were lower than the theoretical distribution. Considering the increase in CFD, the single-cell encapsulation rate showed a greater deviation from the theoretical distribution. This deviation may be because the actual concentration of cells in the high-concentration solution (high *λ*) was relatively low owing to the inevitable cell aggregation and sedimentation, which may have had a side effect on Poisson’s assumption of sparse and independent events. Some observed outliers, including single-cell and multicell encapsulation rates of 21.62% and 5.33% and 22.65% and 4.55%, also supported this judgment. Therefore, the exact relationship between the high *λ* and initial concentrations of cells and the droplet diameter should be established in the future.

It was also found that the A-type chip demonstrated a better approximation to all theoretical distributions. For example, if the flow rates of the dispersed and continuous phases were set to 0.06 mL/h and 0.8 mL/h, respectively, the diameter of the droplets reached 19.42 µm, while the single-cell and multicell encapsulation rates were 24.87% and 4.6%, respectively, which were close to their theoretical values. By establishing the relationship between *λ* and cell concentration, the optimal droplet diameter was calculated, preset by flow rates, and monitored by the proposed method so that the single-cell and multicell encapsulated rates could be stabilized at a desired range. For example, according to the theoretical curve in Figure S2, when *λ* is controlled between 0.26 and 0.27, the single-cell and multicell encapsulation rates can reach >20% and <3%, respectively, ensuring a single-cell rate of >87%. The recognized results of several repeated experiments showed that droplets with diameters between 19 and 21 μm could be obtained, the single-cell encapsulation rate fluctuated between 15.69% and 25.06%, and the multicell encapsulation rate fluctuated by approximately 3%. It was also observed that droplets with a larger diameter, under the same conditions of initial cell density, led to a higher multicell encapsulation rate.

Inertial focusing allows cells to be ordered in a microchannel to break the limits of the Poisson distribution. This study also investigated their effects on cell-encapsulated droplets, and a serpentine structure was added to the C-type chip. Instead of an increase in the single-cell encapsulation rate, a decrease in the multicell encapsulation rate was observed, as shown in Figure 6b. A slight increase in the single-cell droplet rate was suggested when *λ* > 0.5. Considering that the performance of the inertial focusing channel depended on the flow rates and CFD *λ*, it is necessary to strengthen the quality control of experimental conditions, such as improving the uniformity of cell dispersibility and viability and exploring more suitable flow rates for Q_1_ and Q_2_. In the future, a detailed study on the inertial focusing geometry should be performed to draw a more accurate conclusion, while this platform with embedded algorithms will provide an accurate and labor-saving evaluation method for the recognition of cell-encapsulated droplets.

## 5. Conclusions and Perspectives

In this study, we have illustrated passive cell encapsulation in microfluidic droplets as well as the principles and performance of image recognition algorithms. A novel weakly supervised algorithm, WSCNet, was designed to recognize cell-encapsulated droplets from highly adherent droplet images and was systematically verified by different experiments. Compared to classification-based approaches, our method can not only distinguish the droplets encapsulated with different amounts of cells but also locate them without any supervised location information. Reconsidering the weak supervision of droplet-level labels, the WSCNet learns to recognize cell features from the difference between empty and single-cell droplets and then applies the learned knowledge to multicell droplets. Because a multicell droplet encapsulation contains at least two cells inside, the WSCNet can learn to count the cell population from the precise labels and the imprecise labels. In addition, the maximum value in the density map also provides the precise location information of the cell. Unfortunately, if the cells in a multicell encapsulation are highly crowded, which results in a deformed morphological feature, the network may fail to count the cell population and locate each of them.

In addition, the architecture of the proposed counting network only contains seven convolutional layers, which is very tiny and effective. In the future, inertial cell ordering in the dispersed phase should be further investigated to improve the single-cell rate and break Poisson’s distribution [11,12]. We will attempt to extend the proposed method to real-time recognition of video frames. More experiments on cells with different morphologies and the differentiation and enumeration of cell subpopulations should be carried out to make our system available for clinical applications. Considering the integration of a microfluidic chip and imaging algorithm, this system is suitable for applications where rapid analysis of single-cell encapsulation is demanded, such as single-cell sequencing and droplet-based analysis. 

## 6. Patents

The author has a patent pending at Tsinghua University that results from the work reported in this manuscript.

## Figures and Tables

**Table 1:** The distribution of the dataset in different periods for network training and inference.

Stage	Category	Training *				Inference
		All	Train	Validate	Test	
Background		--	28,000	7000	7000	--
Empty		134,188	12,000	3000	3000	>200,000
Single		18,933	12,185	3358	3390	~25,000
Multiple		5830	3821	945	1064	~8000

* The distribution of the dataset in the first period for network training.

## Data Availability

The data that support the findings of this study are available from the corresponding author upon reasonable request. The code is available on GitHub at the following link: https://github.com/Loyage/WSCNet (accessed on 10 July 2023).

## Supplementary Materials

The following supporting information can be downloaded at: https://www.mdpi.com/article/10.3390/bios13080821/s1. Supplementary Note S1. Calculation of the encapsulated cell per droplet by Poisson statistics. Supplementary Note S2. Illustration for adaptive scale template matching (ASTM). Supplementary Note S3. Details for the weakly supervised counting network (WSCNet). Supplementary Note S4. Illustration for nonlinear fitting applied in this study. Figure S1. Optical micrographs of different flow regimes during droplet generation. Figure S2. The encapsulated probability restricted by Poisson’s distribution. Figure S3. Schematic diagram for removing redundant bounding circles. Figure S4. The interaction over union (IoU) of bounding circles. Figure S5. Representative images of the original droplets and the droplet proposals. Figure S6. The quantitative performance of cell localization predicted by the WSCNet. Figure S7. Representative result of the distribution of cell number encapsulated in microfluidic droplets with CPD λ = 0.16. Table S1. Comparative performance of the recognition rate of cell-encapsulated droplets on different methods. Video S1. Representative results of droplet segmentation and cell classification.

## References

[1] Mazutis L., Gilbert J., Ung W.L., Weitz D.A., Griffiths A.D., Heyman J.A. (2013). Single-cell analysis and sorting using droplet-based microfluidics. Nat. Protoc..

[2] Gerard A., Woolfe A., Mottet G., Reichen M., Castrillon C., Menrath V., Ellouze S., Poitou A., Doineau R., Briseno-Roa L. (2020). High-throughput single-cell activity-based screening and sequencing of antibodies using droplet microfluidics. Nat. Biotechnol..

[3] Edd J.F., Di Carlo D., Humphry K.J., Köster S., Irimia D., Weitz D.A., Toner M. (2008). Controlled encapsulation of single-cells into monodisperse picolitre drops. Lab Chip.

[4] Cui P., Wang S. (2018). Application of microfluidic chip technology in pharmaceutical analysis: A review. J. Pharm. Anal..

[5] Zhu P., Wang L. (2017). Passive and active droplet generation with microfluidics: A review. Lab Chip.

[6] Umbanhowar P.B., Prasad V., Weitz D.A. (1999). Monodisperse Emulsion Generation via Drop Break off in a Coflowing Stream. Langmuir.

[7] Anna S.L., Bontoux N., Stone H.A. (2003). Formation of dispersions using “flow focusing” in microchannels. Appl. Phys. Lett..

[8] Thorsen T., Roberts R.W., Arnold F.H., Quake S.R. (2001). Dynamic Pattern Formation in a Vesicle-Generating Microfluidic Device. Phys. Rev. Lett..

[9] Priest C., Herminghaus S., Seemann R. (2006). Generation of monodisperse gel emulsions in a microfluidic device. Appl. Phys. Lett..

[10] Collins D.J., Neild A., Demello A., Liu A.-Q., Ai Y. (2015). The Poisson distribution and beyond: Methods for microfluidic droplet production and single cell encapsulation. Lab Chip.

[11] Kemna E.W.M., Schoeman R.M., Wolbers F., Vermes I., Weitz D.A., Van Den Berg A. (2012). High-yield cell ordering and deterministic cell-in-droplet encapsulation using Dean flow in a curved microchannel. Lab Chip.

[12] Yue X., Fang X., Sun T., Yi J., Kuang X., Guo Q., Wang Y., Gu H., Xu H. (2022). Breaking through the Poisson Distribution: A compact high-efficiency droplet microfluidic system for single-bead encapsulation and digital immunoassay detection. Biosens. Bioelectron..

[13] Lun A.T.L., Riesenfeld S., Andrews T., Dao T.P., Gomes T., Marioni J.C., participants in the 1st Human Cell Atlas Jamboree (2019). EmptyDrops: Distinguishing cells from empty droplets in droplet-based single-cell RNA sequencing data. Genome Biol..

[14] Basu A.S. (2013). Droplet morphometry and velocimetry (DMV): A. video processing software for time-resolved, label-free tracking of droplet parameters. Lab Chip.

[15] Tor S.B., Gañán-Calvo A.M., Chong Z.J., Loh N.H., Nguyen N.-T., Tan S.H. (2016). Automated droplet measurement (ADM): An enhanced video processing software for rapid droplet measurements. Microfluid. Nanofluidics.

[16] Jeong J., Frohberg N.J., Zhou E., Sulchek T., Qiu P. (2018). Accurately tracking single-cell movement trajectories in microfluidic cell sorting devices. PLoS ONE.

[17] Zhu X., Su S., Liu B., Zhu L., Yang W., Gao N., Jing G., Guo Y. (2019). A real-time cosine similarity algorithm method for continuous monitoring of dynamic droplet generation processes. AIP Adv..

[18] Lashkaripour A., Rodriguez C., Mehdipour N., Mardian R., McIntyre D., Ortiz L., Campbell J., Densmore D. (2021). Machine learning enables design automation of microfluidic flow-focusing droplet generation. Nat. Commun..

[19] Zang E., Brandes S., Tovar M., Martin K., Mech F., Horbert P., Henkel T., Figge M.T., Roth M. (2013). Real-time image processing for label-free enrichment of Actinobacteria cultivated in picolitre droplets. Lab Chip.

[20] Svensson C.M., Shvydkiv O., Dietrich S., Mahler L., Weber T., Choudhary M., Tovar M., Figge M.T., Roth M. (2019). Coding of Experimental Conditions in Microfluidic Droplet Assays Using Colored Beads and Machine Learning Supported Image Analysis. Small.

[21] Gardner K., Uddin M.M., Tran L., Pham T., Vanapalli S., Li W. (2022). Deep learning detector for high precision monitoring of cell encapsulation sta-tistics in microfluidic droplet. Lab Chip.

[22] Vo P.Q.N., Husser M.C., Ahmadi F., Sinha H., Shih S.C.C. (2017). Image-based feedback and analysis system for digital microfluidics. Lab Chip.

[23] Vaithiyanathan M., Safa N., Melvin A.T. (2019). FluoroCellTrack: An algorithm for automated analysis of high-throughput droplet microfluidic data. PLoS ONE.

[24] Alcan G., Ghorbani M., Kosar A., Unel M. (2016). A new visual tracking method for the analysis and characterization of jet flow. Flow Meas. Instrum..

[25] Zhao H.F., Zhou J., Gu Y.Y., Ho C.M.B., Tan S.H., Gao Y. Real-Time Computing for Droplet Detection and Recognition. Proceedings of the IEEE International Conference on Real-time Computing and Robotics (IEEE RCAR).

[26] Soldati G., Del Ben F., Brisotto G., Biscontin E., Bulfoni M., Piruska A., Steffan A., Turetta M., Della Mea V. (2018). Microfluidic droplets content classification and analysis through convolutional neural networks in a liquid biopsy workflow. Am. J. Transl. Res..

[27] Xie W.D., Noble J.A., Zisserman A. (2018). Microscopy cell counting and detection with fully convolutional regression networks. Comput. Methods Biomech. Biomed. Eng.-Imaging Vis..

[28] Guo Y., Stein J., Wu G.R., Krishnamurthy A. SAU-Net: A Universal Deep Network for Cell Counting. Proceedings of the 10th ACM International Conference on Bioinformatics, Computational Biology and Health Informatics (ACM-BCB).

[29] Sirinukunwattana K., Raza S.E.A., Tsang Y.-W., Snead D.R.J., Cree I.A., Rajpoot N.M. (2016). Locality Sensitive Deep Learning for Detection and Classification of Nuclei in Routine Colon Cancer Histology Images. IEEE Trans. Med. Imaging.

[30] Montanero J.M., Ganan-Calvo A.M. (2020). Dripping, jetting and tip streaming. Rep. Prog. Phys..

[31] Cubaud T., Mason T.G. (2008). Capillary threads and viscous droplets in square microchannels. Phys. Fluids.

[32] Jensen M.J., Stone H.A., Bruus H. (2006). A numerical study of two-phase Stokes flow in an axisymmetric flow-focusing device. Phys. Fluids.

[33] Cristini V., Tan Y.-C. (2004). Theory and numerical simulation of droplet dynamics in complex flows—A review. Lab Chip.

[34] Zhou C., Yue P., Feng J.J. (2008). Dynamic Simulation of Droplet Interaction and Self-Assembly in a Nematic Liquid Crystal. Langmuir.

[35] Stone H.A., Leal L.G. (1989). Relaxation and breakup of an initially extended drop in an otherwise quiescent fluid. J. Fluid Mech..

[36] Bodla N., Singh B., Chellappa R., Davis L.S. Soft-NMS-Improving Object Detection with One Line of Code. Proceedings of the 16th IEEE International Conference on Computer Vision (ICCV).

[37] Krizhevsky A., Sutskever I., Hinton G.E. (2017). Imagenet classification with deep convolutional neural networks. Commun. ACM.

[38] Szegedy C., Vanhoucke V., Ioffe S., Shlens J., Wojna Z. Rethinking the Inception Architecture for Computer Vision. Proceedings of the 2016 IEEE Conference on Computer Vision and Pattern Recognition (CVPR).

[39] He K.M., Zhang X.Y., Ren S.Q., Sun J. Deep Residual Learning for Image Recognition. Proceedings of the 2016 IEEE Conference on Computer Vision and Pattern Recognition (CVPR).

