# Supplementary material for: WSCNet: Biomedical Image Recognition for Cell Encapsulated Microfluidic Droplets
Source: Biosensors (Basel). 2023 Aug 15;13(8):821. doi: 10.3390/bios13080821 (PMC10452702; doi:10.3390/bios13080821)
Supplement: Supplementary file 1 [file biosensors-13-00821-s001.zip › biosensors-2528063 sup final.pdf]

Supplementary Information

WSCNet: Biomedical Image Recognition for Cell Encapsulated Microfluidic Droplets

Xiao Zhou, Yuanhang Mao, Miao Gu and Zhen Cheng *

Department of Automation, Tsinghua University, Beijing 100084, China; zhouxiao@pjlab.org.cn (X.Z.); maoyh22@mails.tsinghua.edu.cn (Y.M.); gum16@mails.tsinghua.edu.cn (M.G.)

* Correspondence: zcheng@mail.tsinghua.edu.cn

† This paper is an extended version of our paper published in the IEEE 18th International Symposium on Biomedical Imaging (ISBI 2021), Nice, France, 13–16 April 2021.

Table of contents

Supplementary Note 1. Calculation of encapsulated cell per droplet by Poisson statistics.

Supplementary Note 2. Illustration for adaptive scale template matching (ASTM).

Supplementary Note 3. Details for weakly supervised counting network (WSCNet).

Supplementary Note 4. Illustration for nonlinear fitting applied in this study.

Figure S1. Optical micrographs of different flow regimes during droplet generation.

Figure S2. The encapsulated probability restricted by the Poisson's distribution.

Figure S3. Schematic diagram for removing redundant bounding circles.

Figure S4. The interaction over union (IoU) of bounding circles.

Figure S5. Representative images of original droplets and the droplet proposals.

Figure S6. The quantitative performance of cell localization predicted by WSCNet.

Figure S7. Representative result of the distribution of cell number encapsulated in microfluidic droplets with CPD λ = 0.16.

Table S1. Comparative performance of recognition rate of cell encapsulated droplets on different methods.

Video S1. Representative results of droplet segmentation and cell classification.

Supplementary Note 1. Calculation of encapsulated cell per droplet by Poisson statistics

According to following statistical analysis, the single-cell encapsulation rate mainly depends on four factors: the uniformity of droplet generation, the number of generated microdroplets, the number of detected microdroplets, and the number of positive droplets containing cells.

Considering the results of cell detection in the droplet[1], the proportion of positive droplets (droplets containing cells) is

$$\frac{M^d}{N^d} = 1 - \rho(0) \qquad \text{Equation (S.1)}$$

where N^d and M^d are the number of detected microdroplets and positive microdroplets, respectively. According to Equation (S.1), the posteriori value λ of cell per droplet (CPD, cell density divided by droplet volume) can be calculated:

$$\lambda = -ln\ (1 - \frac{M^d}{N^d}) \qquad \text{Equation (S.2)}$$

where N represents the total number of formed microdroplets, then, the number of cells is denoted as K=N*λ. After substituting into equation (S.2), it is rewritten as:

$$K = -Nln\ (1 - \frac{M^d}{N^d}) \qquad \text{Equation (S.3)}$$

Therefore, the quantitative cell counting depends on four elements: uniformity of the formation process of microdroplets (which ensures that the cell number in a microdroplet meets Poisson distribution), the number of generated microdroplets N, detected microdroplets N^d and its positive microdroplets M^d. The first two elements are related to the formation process of microdroplets and the last two elements are related to the detection process of microdroplets.

When λ=1, the single-encapsulated rate reached the largest 36.8%, at this time, the multi-encapsulated rate and the single-cell rate is about 26% and 58%, respectively. If λ=0.1, the single-cell, single-encapsulated, and multi-encapsulated rates are 95%, 9%, and 0.5%, respectively. When λ is between 0.26 and 0.27, the single-encapsulated rate is more than 20% and the multi-encapsulated rate is less than 3%, as shown in Fig. S2. Consequently, this passive dilution approach is simple but wasteful, as it generates a lot of empty droplets since the single-encapsulated probability is low. If there is a need to encapsulate two distinct cell types in the same droplet, or a cell and a bead, for example, the probability of having one droplet with only one of each entity falls to 13.5% and generates even more waste due to the higher dilution.

Supplementary Note 2. Illustration for adaptive scale template matching (ASTM)

Since the static droplets sustain certain circularity despite the mutual adhesion in images, it is reasonable to use bounding circles instead of a sophisticated mask to describe a droplet. Specifically, the bounding circle of the *i*th droplet is denoted by $C_i = [x_i, y_i, r_i]$, where $[x_i, y_i]$represents the center coordinates of C_i in the image and R_i suggests its radius. As a result, for the highly adherent droplet images, we must locate the centers of all droplet proposals and quantify their radiuses.

Firstly, the Otsu threshold segmentation algorithm is employed to subtract the irrelevant background and extract the droplet foreground, which is almost fully connected as shown in Fig. 2b. To process the binary foreground, the ASTM algorithm is proposed to generate droplet proposals. Specifically, assuming the binary template is noted by $\mathcal{T}$ and its initial size is $r \times r$, the matching response of each pixel in the foreground can be computed by:

$$\mathcal{D}(x,y) = \sum_{a,b \in [0,r)} \mathcal{T}(a,b)\mathcal{F}\left(x+a-\frac{r}{2}, y+b-\frac{r}{2}\right) \quad \text{Equation (S.4)}$$

Where $\mathcal{F}$ denotes the binary foreground image, the bounding circle of the *i*th droplet is denoted by $C_i = [x_i, y_i, r_i]$ and $[x_i, y_i]$ represents the center of *i*th droplet and r_i suggests its radius.

Intuitively, the matching response *D(x,y)* of a droplet center in the foreground will be a local maximum when the scale of the template equals the droplet diameter. Therefore, droplet proposals can be located by the greedy search of all local maxima. Nevertheless, the diameter of droplets varies due to unpredicted fusion. A small template can locate small droplets, but it yields multiple inaccurate response maxima for large droplets and vice versa. It is necessary to elaborate an adaptive scale circular template to find all droplets with various diameters.

Supposing that the radius of the current circular template is r_{cur}, after matching the template with the binary foreground image, the response of each pixel indicates the probability to be a droplet proposal. Accordingly, the pixel whose matching response is higher than a predefined threshold σ will be marked as the center of a droplet proposal and the current template scale suggests the diameter of the droplet proposal. In contrast, if all the matching responses are less than σ, suggesting that all the droplets in the image are smaller than a circle with a radius r_{cur}, the scale of the current template must shrink adaptively.

It can be implied from Equation 1 that the matching response *D(x,y)*, varying from 0 to 1, is essentially the ratio of the foreground area inside the template to the full template area. Consequently, the maximum of matching responses $\mathcal{D}_{\max}$ corresponds to the largest foreground area covered by the template, which can be computed by $\pi r_{cur}^2 \mathcal{D}_{\max}$. Intuitively, the shrinking template radius for the next time matching can be provided by:

$$r_{next} = \sqrt{\mathcal{D}_{\max}}\, r_{cur} \quad \text{Equation (S.5)}$$

In the implementation, we simply need to set the upper and lower bound of the template radius to perform a limited number of searches. Since overlapping bounding circles might occur in one true droplet, non-maximum suppression (NMS)[2] is employed to remove redundant bounding circles, as shown in Fig. S3.

Supplementary Note 3. Details for weakly supervised counting network (WSCNet)

The second stage is the recognition of N-cells droplet encapsulation from the droplet proposals provided in the last section. Most studies train deep convolutional neural networks to simplify this issue by classifying the droplet proposals into four categories: false positive, empty droplet, single-cell, and multicell encapsulations. Classification-based algorithms, including AlexNet[3], VGG16, Inception V3[4], ResNet18[5] and ResNet34[5], were applied as benchmarks to simplify this task by classifying the proposals into four categories: background, empty, single-cell, and multicell encapsulations. Unfortunately, their training time and resource consumption will greatly increase as the network deepens. Moreover, lacking interpretability, the classification task can neither count the specific number of cells inside each droplet proposal nor predict the location of each cell.

As a result, counting the cell population provides an explainable solution for the recognition of N-cells droplet encapsulation. To avoid tedious manual cell-level annotation, only three droplet-level labels, including empty, single-cell, and multicell capsulation, are adopted in this work. We develop a WSCNet to estimate the number of cells inside each droplet proposal and predict the location of each cell.

The WSCNet consists of two branches: classification and counting, as shown in Fig. 2. The classification branch serves as a filter to remove false positives in droplet proposals, and the counting branch counts and locates cells inside each droplet. The output of the counting branch is a single channel density map of cells. The integral and the local maxima of the density map indicate the number and the location of cells, respectively.

Adopting the conventional cross entropy as the loss function, the classification branch aims to provide a predicted label (droplet or false positive) for each droplet proposal. The counting branch, however, employs the mean square error between the true counting label and the counting prediction as its loss function:

$$L_{count} = \|f(\mathcal{D}) - y\|^2 \qquad \text{Equation (S.6)}$$

Where, $\mathcal{Y}$ suggests the supervision, *i.e.* the true counting label, $\mathcal{D}$ and $f(\mathcal{D})$ denote the output density map and its counting prediction, respectively. Generally, the counting prediction $f(\mathcal{D})$ can be obtained by the global sum pooling of the density map. Nevertheless, the weak supervision for the network provides only two precise counting labels (0 for empty, 1 for single-cell) and one imprecise label (>1 for multicell). Reasonably, we assume that the multicell droplet encapsulation contains at least 2 cells, and then quantify its label as 2 meanwhile truncating its counting prediction to 2. Considering that the truncation function will degenerate to a constant resulting in a vanishing gradient issue, it is necessary to add a small gradient for the truncation function, which can thus be formulated by:

$$f(\mathcal{D}) = \begin{cases} \mathcal{D}_{sum} & \mathcal{D}_{sum} < 2 \\ 2 + \gamma \mathcal{D}_{sum} & \mathcal{D}_{sum} \geqslant 2 \end{cases}$$

Equation (S.7)

Where, $\mathcal{D}_{sum}$ indicates the integral of the density map, which can be obtained by global sum pooling:

$$\mathcal{D}_{sum} = \sum_{i,j} \mathcal{D}(i,j)$$

Equation (S.8)

γ represents a small constant, which provides a small gradient that avoids the vanishing gradient issue.

It is a reasonable assumption that the density value of each pixel is less than 1 since the density map has the same resolution as the input image. Accordingly, a regularization performed on each density value is added to the loss function that can avoid the overestimation of counting prediction caused by the truncation. Noticing that the limitation of each value in the density map is equivalent to the restriction of the max density value, the regularization can be given:

$$\psi(\mathcal{D}) = \begin{cases} \mathcal{D}_{max} - 1 & \mathcal{D}_{max} > 1 \\ 0 & \mathcal{D}_{max} \leqslant 1 \end{cases}$$

Equation (S.9)

Where, $\mathcal{D}_{\max}$ represents the max value in the density map, which can be obtained by global max pooling.

So far, the loss function of the counting branch can be given by:

$$L_{count} = \|f(\mathcal{D}) - y\|^2 + \psi(\mathcal{D})$$

Equation (S.10)

Where, $f(\mathcal{D})$ and $\psi(\mathcal{D})$ are shown in Eq. (6) and Eq. (7), respectively. The loss function of the whole network containing the classification and the counting branches is given with a weight of λ:

$$L = \lambda L_{count} + (1 - \lambda)\, L_{class}$$

Equation (S.11)

Where, L_{class} denotes the loss function of cross entropy for the classification branch.

In the forward reasoning stage, the classification branch provides a predicted label and the counting branch outputs a density map with the same resolution as the input image. The density map is valid only when the predicted label is "droplet". Moreover, the integral of the density map $\mathcal{D}_{sum}$suggests the cell population, and the first $\lfloor \mathcal{D}_{sum} + 0.5 \rfloor$ maximums in the density map indicate the locations of all cells. To compare with other classification-based approaches, the droplets can be reclassified into three categories (empty, single-cell, and multicell encapsulations) according to their cell numbers:

$$id = \underset{k \in \{0,1,2\}}{argmin} \, (\mathcal{D}_{sum} - k)^2$$

Equation (S.12)

Supplementary Note 4. Illustration for nonlinear fitting applied in this study

Assume the average number of cells in each microdroplet CPD is λ which follows Poisson distribution $\rho(\lambda)$; then, the probability of k cells in one microdroplet can be calculated according to:

$$\rho(k) = \frac{\lambda^k}{k\,!} exp(-\lambda) \quad \text{Equation (S.13)}$$

The number of cells encapsulated per droplet in passive methods is randomly restricted by Poisson statistics. Therefore, multiple statistical results were first fitted to the Poisson's theoretical distributions (as shown in Figure S3) with error analysis conducted by the Residual Sum of Squares (RSS) test. Their corresponding RSS and probability distribution of single-cell (N=1), multi-cell (N>1) encapsulation rate, empty droplet rate (N=0), and single-cell rate (N=1 / N≥1, the proportion of droplets containing exactly one cell to that containing cells) is plotted in Figure 6.

However, in the experimental tests, there may be a bias in the estimation of droplet diameter and initial cell concentration, which leads to a bias in the calculation of CPD λ. In addition, different nonlinear fitting curves were applied for the approximated fitting of the theoretical distributions on encapsulation rates and the single-cell rate, which can be written as follows:

$$\rho(1) = A * \lambda * exp(-\lambda * B) + C \quad \text{Equation (S.14)}$$

$$\rho(> 1) = 1 - A * \lambda * exp(-B * \lambda) - C * exp(-\lambda) + D \quad \text{Equation (S.15)}$$

$$\rho(0) = A * exp(-\lambda * B) \ + \ C \quad \text{Equation (S.16)}$$

$$\frac{\rho(1)}{\rho(\geq 1)} = \frac{A*\lambda*exp(-B*\lambda)}{(1-C*exp(-\lambda))} + D \quad \text{Equation (S.17)}$$

where A, B, C, and D, are parameters to be fitted with a fixed initialization. The Equation (S.14)~(S.17) is used for the nonlinear fitting of single-cell (N=1), multi-cell (N>1) encapsulation rate, empty droplet rate (N=0), and single-cell rate (N=1 / N≥1), respectively. Levenberg Marquardt (integrated in OriginPro 2021) is used as an iteration algorithm with 400 max iterations and 1E-9 tolerance. Fitted curves with the corresponding 95% confidence bands and predication bands are plotted.

Supplementary Figure

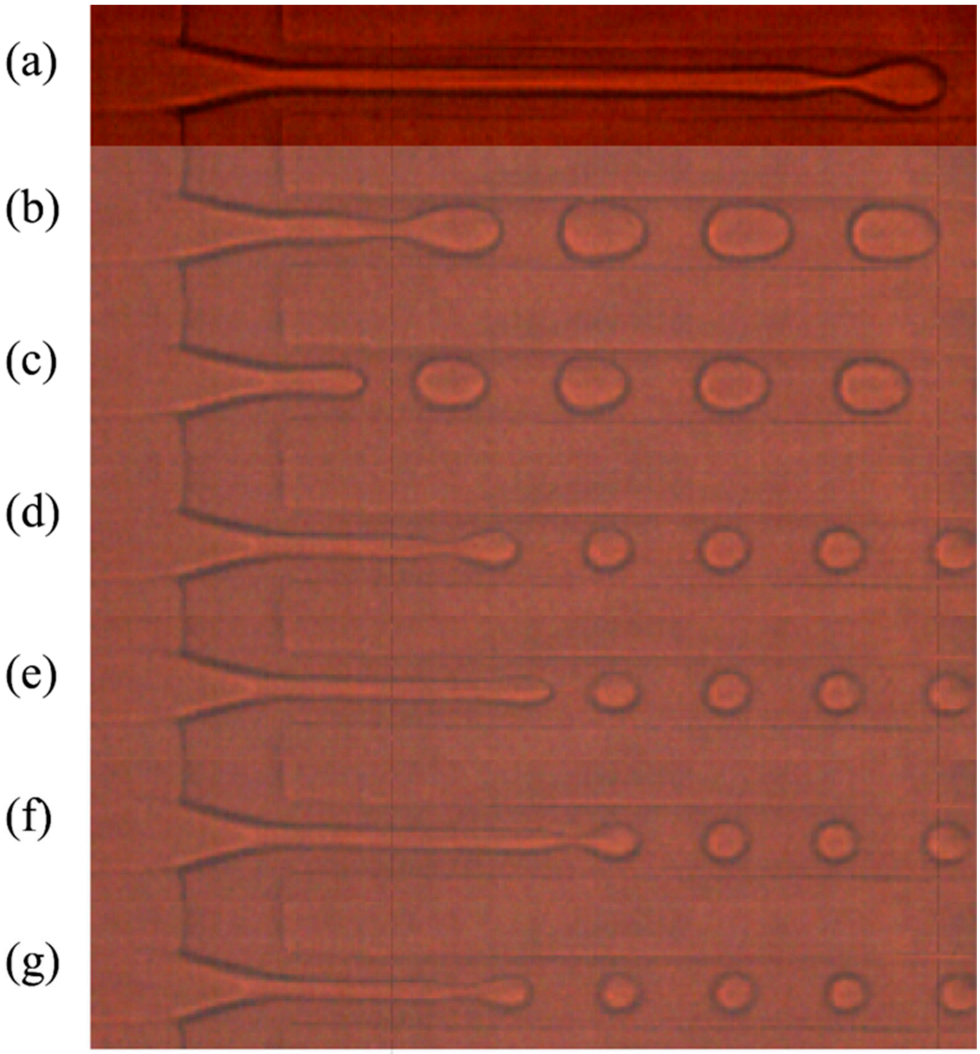

Figure S1. Optical micrographs of different flow regimes during droplet generation. (A) the tubing regime, (B, D-G) the jetting regime, and (C) the dripping regime. The flow rate of the dispersed phase Q_1 was fixed to 0.2mL/h, while the flow rates of the continuous phase Q_2 are set to 0.2, 0.25, 0.35, 0.6, 0.65, 0.7, 0.8 mL/h, respectively, from top to bottom. When the generated droplets were located within two channel widths away from the cross-section, a dripping mode is considered. Similarly, a jetting mode or a tubing mode is considered if droplets appeared stably within 2-10 times of channel width or more than 10 times of channel width, respectively. Transition means flow oscillates periodically between the tubing and the jetting modes. These micrographs were collected by a high-speed camera which is not adopted in the subsequent experiments.

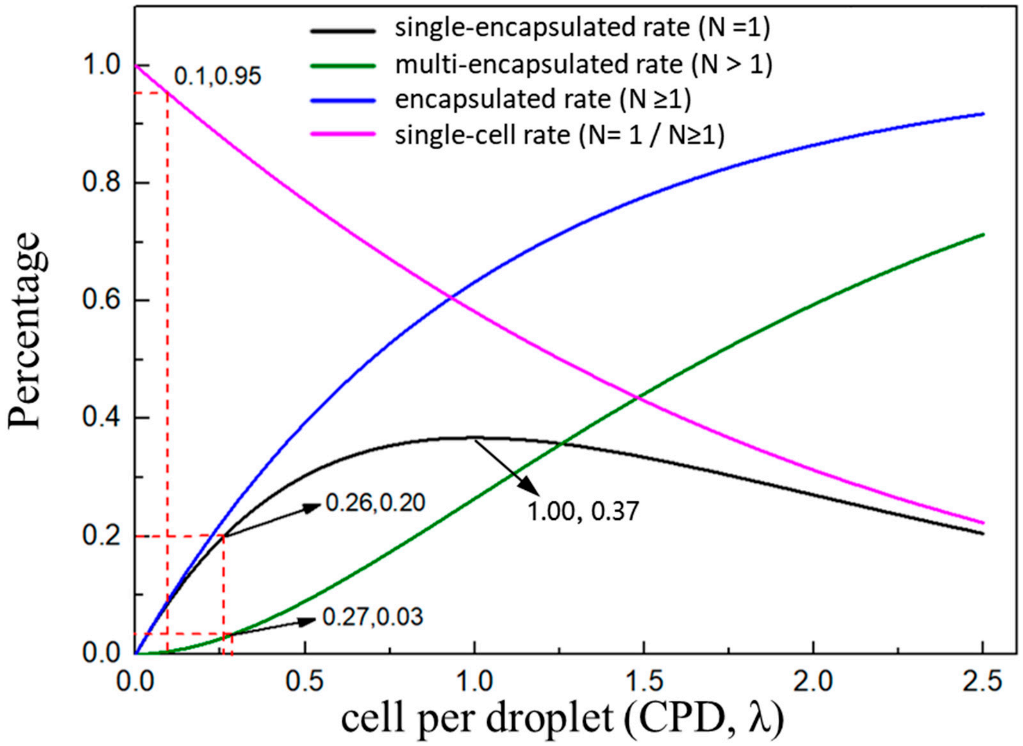

Figure S2. The encapsulated probability restricted by the Poison's distribution. The number of cells encapsulated per droplet in passive methods is randomly restricted by Poisson statistics. The probability distribution of single-encapsulated (N =1), multi-encapsulated (N > 1), encapsulated (N ≥1), and single-cell rate (N=1 / N≥1, the proportion of droplets containing exactly one cell to that containing cells) is plotted with different colors. Supposing the number of cells encapsulated per droplet is X, X follows Poisson distribution X ~ P (λ).

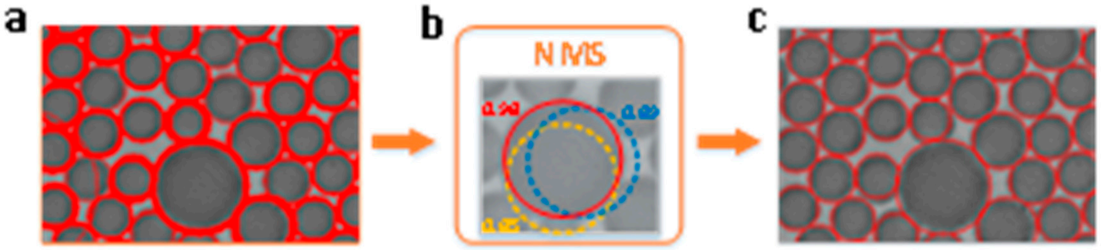

Figure S3. Schematic diagram for removing redundant bounding circles. (A) Overlapping bounding circles. (B) Non-maximum suppression (NMS) is adopted to remove overlapping droplet proposals with lower matching responses. (C) Final prediction of droplet proposals.

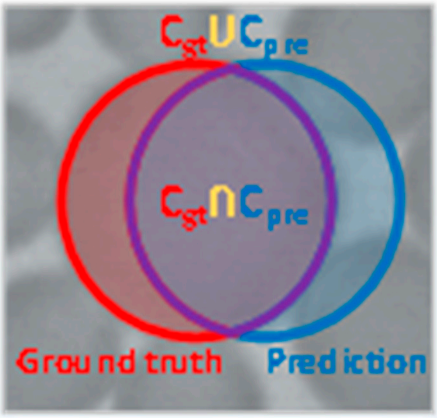

Figure S4. The interaction over union (IoU) of bounding circles. The ground truth and prediction of droplet is marked as Cgt and Cpre, respectively. IoU, also known as the Jaccard index, is the most popular evaluation metric for tasks such as segmentation and object detection. IoU is then computed as the ratio of intersection area (purple) to the union area (both red and blue).

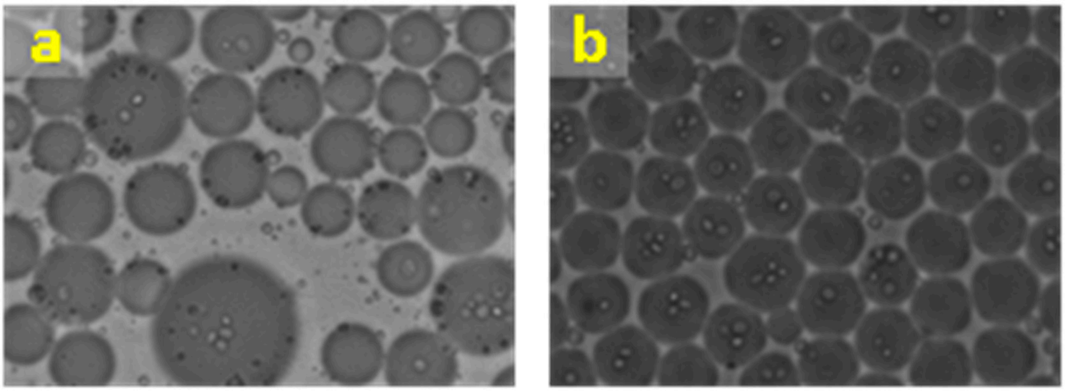

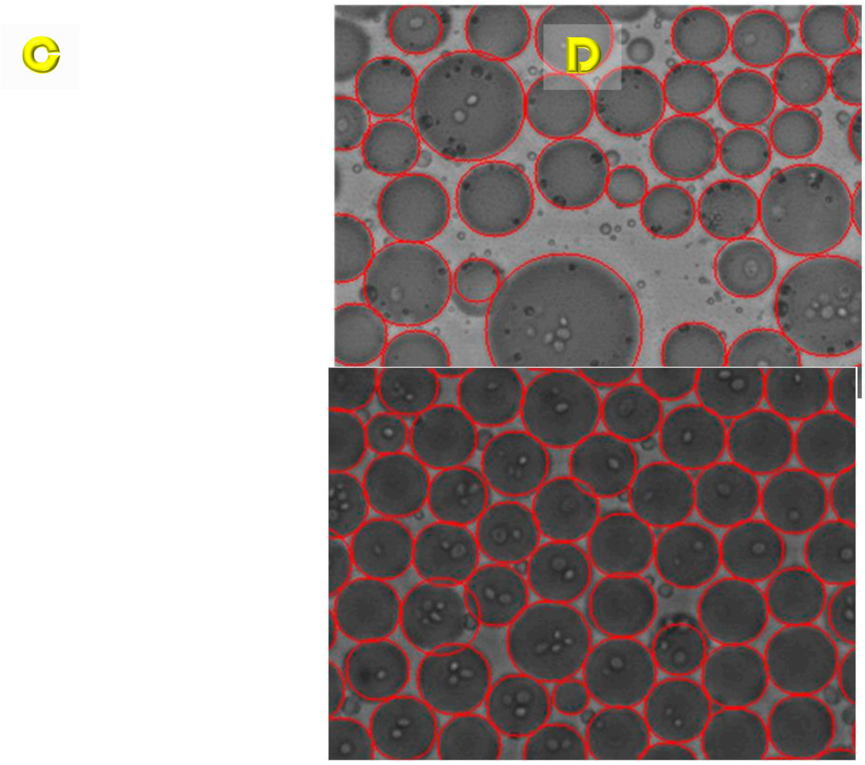

Figure S5. Representative images of original droplets and the droplet proposals. (A) Different droplets in various diameters. (B) Highly adherent and crowded droplets. (C-D) Predicted droplet proposals with red bounding circles in multi-scale and highly adherent droplet images. This paper uses static microscopic images to recognize droplets with diverse cell numbers inside while facing the challenges of different droplet scales and high adhesiveness.

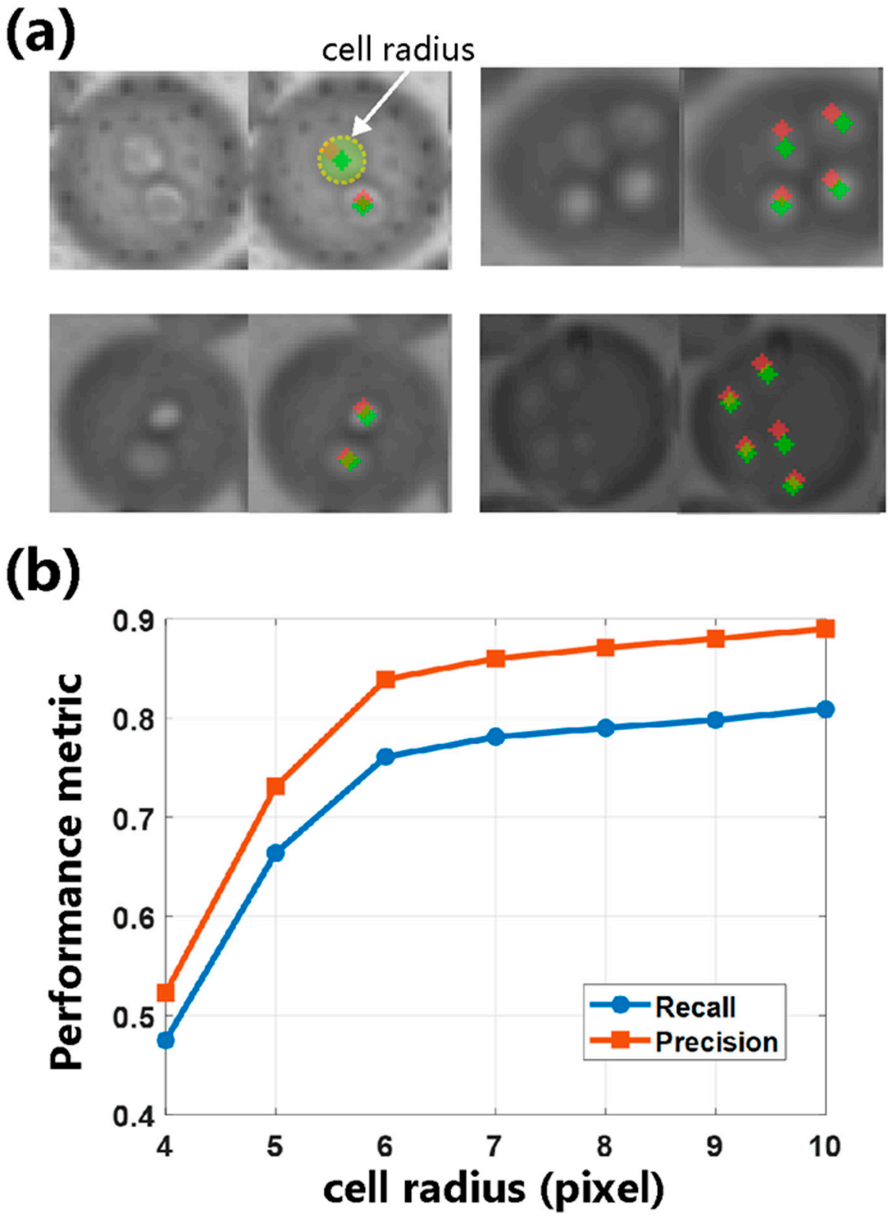

Figure S6. The quantitative performance of cell localization predicted by WSCNet. (A) Examples of predicted micrographs of droplets encapsulated cells. The ground truth of each cell in the test dataset of multicell encapsulation is marked as green points, which is not applied in the training procedure, and the predicted location of each cell is marked as red points. A circular mask with an x-pixel radius centered at each annotated cell centroid is regarded as a valid area. (B) Cell location performance under different cell radii. The recall and precision curves are evaluated at different cell radii ranging from 4 to 10 pixels.

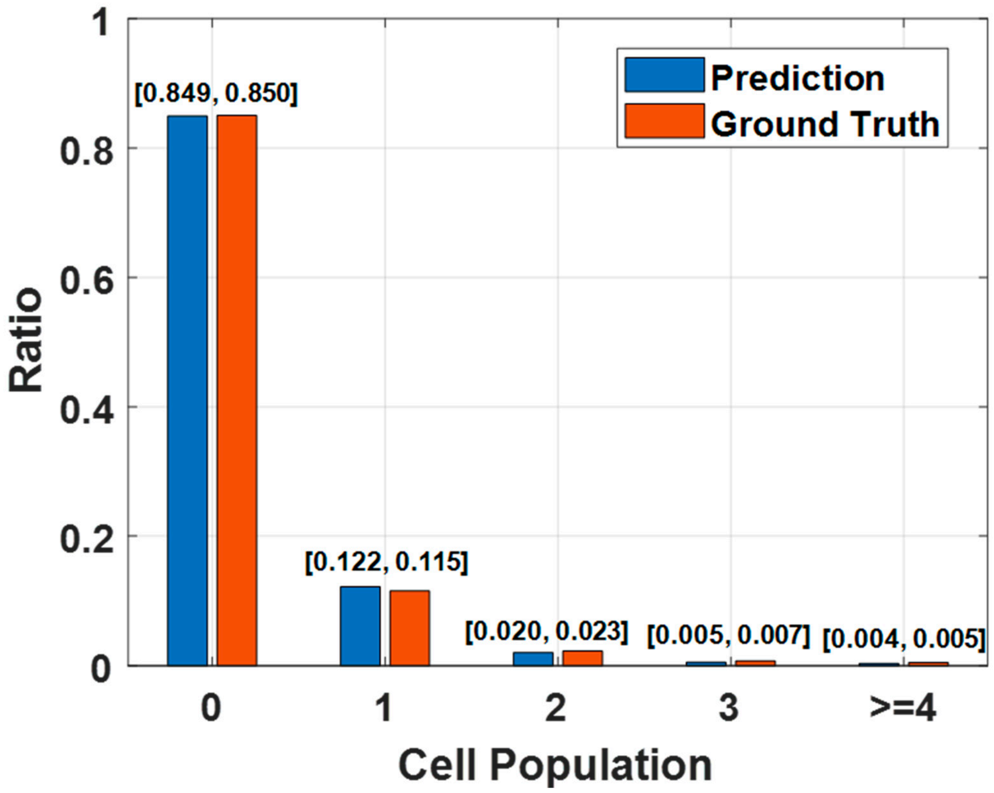

Figure S7. Representative result of cell amount distribution encapsulated in microfluidic droplets with CPD λ = 0.16. The cell encapsulated in droplets were counted and localized by our proposed method while the encapsulated cell amount is obtained (marked in prediction) and compared with the manual annotation (marked in ground truth).

Supplementary Table

Table S1. Comparative performance of recognition rate of cell encapsulated droplets on different methods.

Method \ Recall	Background	Empty	Single	Multiple	Model size (MB)	Training time (min)
**AlexNet**	0.998	0.984	0.961	0.897	217.0	231
**VGG16**	0.999	**0.996**	0.972	0.916	512.3	1574
**InceptionV3**	0.999	0.993	0.972	0.925	84.6	1546
**Resnet18**	0.999	0.987	0.961	0.895	42.7	**153**
**Resnet34**	0.999	0.984	0.966	0.921	81.3	250
**Ours**	**0.999**	0.987	**0.973**	**0.938**	**11.7**	168

References

[1] Collins, D.J., A. Neild, A. deMello, *et al.*, *The Poisson distribution and beyond: methods for microfluidic droplet production and single cell encapsulation.* Lab on a Chip, 2015. **15**(17): p. 3439-3459.

[2] Bodla, N., B. Singh, R. Chellappa, *et al. Soft-NMS - Improving Object Detection With One Line of Code*. in *16th IEEE International Conference on Computer Vision (ICCV)*. 2017. Venice, ITALY: Ieee.

[3] Krizhevsky, A., I. Sutskever, and G.E. Hinton, *ImageNet classification with deep convolutional neural networks.* Communications of the ACM, 2017. **60**(6): p. 84-90.

[4] Szegedy, C., V. Vanhoucke, S. Ioffe, *et al. Rethinking the Inception Architecture for Computer Vision*. in *2016 IEEE Conference on Computer Vision and Pattern Recognition (CVPR)*. 2016. Seattle, WA: Ieee.

[5] He, K.M., X.Y. Zhang, S.Q. Ren, *et al. Deep Residual Learning for Image Recognition*. in *2016 IEEE Conference on Computer Vision and Pattern Recognition (CVPR)*. 2016. Seattle, WA: Ieee.
